# The Role of Protein Kinase C During the Differentiation of Stem and Precursor Cells into Tissue Cells

**DOI:** 10.3390/biomedicines12122735

**Published:** 2024-11-29

**Authors:** Oliver Pieles, Christian Morsczeck

**Affiliations:** Department of Oral and Maxillofacial Surgery, University Hospital Regensburg, Franz-Josef-Strauss-Allee 11, 93053 Regensburg, Germany; oliver.pieles@vkl.uni-regensburg.de

**Keywords:** stem cells, precursor cells, differentiation, development, protein kinase C, osteogenic differentiation, adipogenic differentiation, chondrogenic differentiation, neuronal differentiation

## Abstract

Protein kinase C (PKC) plays an essential role during many biological processes including development from early embryonic stages until the terminal differentiation of specialized cells. This review summarizes the current knowledge about the involvement of PKC in molecular processes during the differentiation of stem/precursor cells into tissue cells with a particular focus on osteogenic, adipogenic, chondrogenic and neuronal differentiation by using a comprehensive approach. Interestingly, studies examining the overall role of PKC, or one of its three isoform groups (classical, novel and atypical PKCs), often showed controversial results. A discrete observation of distinct isoforms demonstrated that the impact on differentiation differs highly between the isoforms, and that during a certain process, the influence of only some isoforms is crucial, while others are less important. In particular, PKCβ inhibits, and PKCδ strongly supports osteogenesis, whereas it is the other way around for adipogenesis. PKCε is another isoform that overwhelmingly supports adipogenic differentiation. In addition, PKCα plays an important role in chondrogenesis, while neuronal differentiation has been positively associated with numerous isoforms including classical, novel and atypical PKCs. In a cellular context, various upstream mediators, like the canonical and non-canonical Wnt pathways, endogenously control PKC activity and thus, their activity interferes with the influence of PKC on differentiation. Downstream of PKC, several proteins and pathways build the molecular bridge between the enzyme and the control of differentiation, of which only a few have been well characterized so far. In this context, PKC also cooperates with other kinases like Akt or protein kinase A (PKA). Furthermore, PKC is capable of directly phosphorylating transcription factors with pivotal function for a certain developmental process. Ultimately, profound knowledge about the role of distinct PKC isoforms and the involved signaling pathways during differentiation constitutes a promising tool to improve the use of stem cells in regenerative therapies by precisely manipulating the activity of PKC or downstream effectors.

## 1. Introduction

Protein kinase C (PKC) is an enzyme that is involved in many biological processes. Since PKC was first described in 1977 [1], much research has been conducted in order to unravel the physiological and pathological implications of the kinase. Developmental processes were one major field of interest, and today, it has been established that PKC plays an essential role during development from early embryonic stages until the formation of adult tissues [2,3]. Hence, it stands to reason that, on a cellular level, the kinase affects the differentiation of stem cells into specialized tissue cells, which also applies for in vitro cultures. However, its function in these processes is very complex and varies highly between different PKC isoforms. Moreover, plenty of cellular targets are affected, which makes it difficult to determine the contribution of distinct downstream pathways [4]. In recent years, stem cells have gained increasing attention as promising tools for regenerative therapies [5]. Thus, a solid understanding of the molecular mechanisms during differentiation would certainly help to provide the best possible preconditions for their safe and directed clinical use. This review comprehensively summarizes the current knowledge and latest research on how PKC is involved in the differentiation of tissue cells.

**Figure 1:**
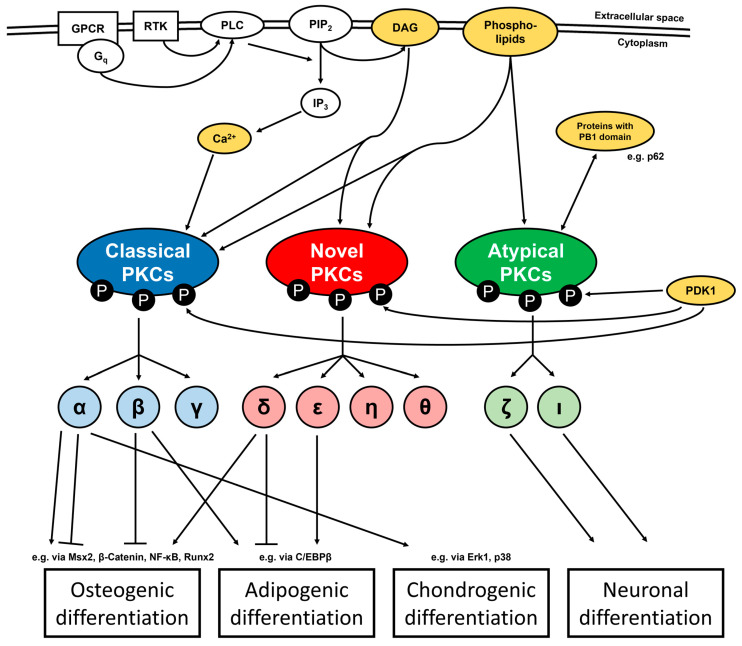
Model of the molecular mechanisms that activate the different PKC classes and their most important implications for differentiation into tissue cells. Each PKC class is illustrated in its own color (with the single isoforms in lighter shades). Substances which directly activate or regulate PKC are colored yellow. For further explanations see text.

PKC represents a family of serine/threonine kinases, which in humans consists of ten different isoforms that can be distinguished into classical isoforms (α, β1, β2, γ; note: β1 and β2 originate from the same gene by alternative splicing and both isoforms are often considered together), novel isoforms (δ, ε, η, θ) and atypical isoforms (ζ, ι) with different activation mechanisms for each of the groups [4,6]. The activation of classical isoforms depends on diacylglycerol (DAG) and phospholipids—especially phosphatidylserine—in the plasma membrane, as well as intracellular Ca^2+^ ions. By contrast, novel isoforms only require DAG and phospholipids, while atypical isoforms are regulated by phospholipids and protein–protein interactions [4,7,8]. The most prominent activation mechanism—at least for the classical and novel isoforms—involves the activation of phospholipase C (PLC) by G protein-coupled receptors (GPCRs) with a G_q_ protein or by receptor tyrosine kinases (RTKs). These in turn raise the concentrations of DAG in the plasma membrane and, via the production of inositol 1,4,5-trisphosphate (IP_3_), Ca^2+^ ions in the cytoplasm [9,10,11]. Moreover, an important prerequisite for PKC activation is the abundance of three distinct phosphorylations at the amino acid residues T500, T641 and S660 (corresponding to the positions in PKCβ2; positions slightly vary between the isoforms), which are mediated by the kinase 3-phosphoinositide-dependent protein kinase-1 (PDK1) and by autophosphorylation [12,13,14]. Figure 1 schematically illustrates the molecular mechanisms that regulate the activity of the different PKC isoform classes as well as their principial implications during the differentiation of tissue cells, which are presented in detail in the following chapters.

**Figure 2:**
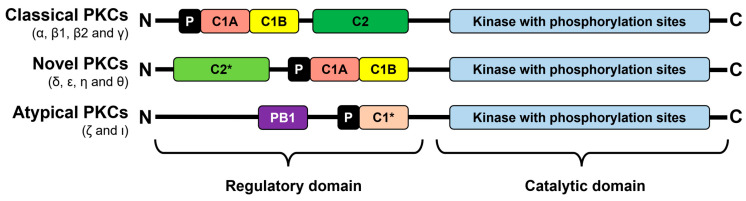
Schematic structure of the different PKC isoforms. The figure schematically shows the structure of the different groups of PKC isoforms with the most important domains. Equal elements are illustrated in the same color. Group-specific variants of domains lacking the ability of binding to the corresponding cofactors are colored in a lighter shade and indicated with asterisks. P indicates the pseudosubstrate domain. For further explanations see text.

The reason for the different activation requirements is based on the structural diversities between the isoform groups, which are depicted in Figure 2. The basic structure of all isoforms consists of a regulatory N-terminal domain, which is responsible for interaction with cofactors, and a catalytic C-terminal domain. The regulatory N-terminal domain of classical PKCs contains two different C1 domains for DAG binding and a C2 domain, which binds Ca^2+^ ions. By contrast, novel and atypical isoforms partially lack these domains or only include variants without the ability for cofactor binding. Instead, atypical PKCs contain a Phox and Bem1 (PB1) domain, which facilitates interactions with other PB1 domain-containing proteins that, in turn, are involved in various biological pathways. For example, PKCζ has been shown to interact with the protein p62, which is known for binding ubiquitinated proteins scheduled to undergo autophagy [15,16,17,18]. Before the binding of the corresponding second messengers, PKC is kept in an inactive state by an autoinhibitory pseudosubstrate, which is part of the regulatory domain of all isoforms and blocks the substrate binding site until activation [19,20].

## 2. Regulation of Biological Processes by PKC

Many substrates are phosphorylated by PKC with several overlaps in the substrate specificity between different isoforms [4]. Hence, the role of PKC in a certain biological process can be very diverse and ambiguous. A striking example is the regulation of cell cycle by PKC. Usually, PKC is positively associated with cell division and especially with the proliferation of tumor cells [21,22,23], while pharmacological inhibition of PKC has been reported to attenuate the cell cycle in several studies [24,25,26]. However, an increasing number of publications have reported a negative impact of PKC on cell proliferation, which suggests that the kinase might play an important role as tumor suppressor under some circumstances [27,28,29,30]. Observation of only selected isoforms showed that especially classical PKCs were contrarily associated with cell proliferation, since studies reported both positive [31,32] and negative [33] impacts on the cell cycle. This might be particularly due to PKCα, which either supports [34,35,36] or inhibits [37,38] proliferation, whereas PKCβ1/2 and PKCγ mainly promote cell division [31,32,39]. The reason for this can be found in the wide range of proteins that interact with PKCα, which includes cell cycle proteins like cyclin D1 as well as cell cycle inhibitors like p53, p21 and p27 [40,41,42,43]. In addition, PKCδ and atypical PKCs are overwhelmingly positively associated with cell proliferation [44,45,46,47,48]. Moreover, PKC is involved in apoptotic processes as the kinase can be cleaved by caspases and, vice versa, regulate apoptosis itself [49,50,51], which further affects the viability and growth rates of cells.

Another crucial aspect regulated by PKC is cellular energy metabolism. For example, Heathcote et al. showed that PKC can phosphorylate the enzyme AMP-activated protein kinase (AMPK), which is usually activated by cellular energy scarcity and mainly stimulates catabolic processes resulting in higher ATP levels. The described phosphorylation, which is supposed to be primarily exerted by classical isoforms, occurs at Ser487 of the catalytic subunit isoform α1 of AMPK and leads to a reduction in its activity [52]. Thus, (classical) PKC isoforms might also indirectly affect metabolic downstream processes. Indeed, studies have already shown that PKC regulates glycolysis and autophagy, which are both processes that are affected by AMPK [53,54,55]. Moreover, several PKC isoforms from all three groups were shown to regulate the energy metabolism of mitochondria, which ultimately affects their ability to produce ATP [56,57,58,59]. Again, the impact of the kinase highly differs between the isoforms even in the same group as—for example—PKCδ has been reported to support mitochondrial function, whereas PKCε has been shown to act contrarily [56,57].

## 3. Impact of PKC on General Stem Cell Properties

During development, specialized tissue cells emerge from different types of stem cells, which are characterized by their differentiation potential as well as their ability to self-renew. Depending on the number of possible cell types that can arise, stem cells are usually classified to be either totipotent (zygote and cells of early blastomere stages), pluripotent (embryonic stem cells and induced pluripotent stem cells) or multipotent (e.g., mesenchymal stem cells, MSCs) [60,61]. In addition, progenitor cells, which are already determined to develop into a certain cell type, are categorized as unipotent [62]. MSCs represent a widely used tool in stem cell research. A huge advantage, besides their multipotency, is that they are present not only in developing but also in adult tissues like in the bone marrow (bone marrow-derived mesenchymal stem cells, BMMSCs) or adipose tissue (adipose-derived stem cells, ADSCs), which makes them relatively easy to acquire. After isolation, established protocols allow for expansion in vitro, while retaining their multipotency for several passages [61,63]. Typically, MSCs possess the ability of so-called trilineage differentiation into either osteoblasts, adipocytes or chondrocytes [64]. However, some types of MSCs can also trans-differentiate in vitro into several other cells like neurons or myocytes [61]. In some cases, even progenitor cells that are already pre-differentiated into a certain direction can be nudged towards another differentiation pathway in vitro by appropriate stimuli [65,66]. Because of their properties, stem and—at least partially—progenitor cells represent a very promising tool for use in regenerative therapies [67].

According to the examples of cell cycle and metabolic regulation, a similarly complex role of PKC for the characteristics of stem cells can be assumed. For instance, a study by Feng et al. suggested that PKCδ in particular might be important for stem cells to acquire a mesenchymal phenotype during early embryonic development [68]. In addition, the novel isoforms PKCε and PKCθ might be involved in gaining mesenchymal properties, as their activity has been associated with the epithelial–mesenchymal transition of cells—a mechanism which is mainly observed during tumorigenesis, but probably possesses similarities with the molecular mechanisms during the fate decision of stem cells [69,70]. Moreover, energy metabolism, which is highly regulated by PKC (see above), influences the pluripotency of stem cells. These preferably rely on glycolysis while retaining an undifferentiated state, whereas increased mitochondrial activity has been associated with cellular differentiation and the loss of pluripotency [71,72]. Another important property of stem cells is their ability to migrate to distinct sites where they are required [73]. Experiments showed that PKCε supports the migration of MSCs [74] and the isoforms PKCα and PKCδ have been associated with MSC migration after induction by interleukin-1β in a study by Lin et al. [75]. These numerous examples show that especially novel PKCs might possess important roles for the general properties of stem cells, which are required irrespective of their functions as specialized tissue cells later. However, the role of distinct PKC isoforms changes regarding differentiation into specific cell types, which will be discussed in the following chapters containing the essential results from studies carried out with various stem and precursor cells.

## 4. Role of PKC During Osteogenic Differentiation

### 4.1. General Influence of PKC on Osteogenesis

While, to date, not a huge number of studies have investigated the distinct roles of PKC during the differentiation of stem and precursor cells, it is though best explored for the process of osteogenesis. However, as with its involvement in proliferation, the results of different studies are partially inconsistent. This is not surprising since proliferation and (osteogenic) differentiation are usually considered as consecutive, but mutually exclusive processes [76,77].

Another similarity to cell cycle regulation is the observation that most published data assume an overall supportive role of PKC for osteogenesis [78,79,80,81,82,83]. Hence, the activity of the kinase appears to be crucial for the whole developmental process of bone formation from residing stem cells to a bulk of differentiated, matrix-producing osteoblasts. Moreover, PKC-dependent stimulation of stem cell proliferation might even be a prerequisite for subsequent osteogenesis. Indeed, this assumption is in line with studies showing that several pathways that support stem cell proliferation also enhance osteogenic differentiation of MSCs [84,85,86,87]. Presumably, osteogenesis can only start from a sufficient number of stem/precursor cells, which might only be achieved by an adequate proliferation rate priorly.

In contrast, Jeong et al. showed the opposite in experiments with murine C2C12 cells, which represent a myoblast cell line that is capable of trans-differentiating into osteoblasts in vitro following stimulation with bone morphogenetic protein 2 (BMP2). In this model, PKC was shown to inhibit osteogenic differentiation by stabilizing the transcription factor Msh homeobox 2 (Msx2), which in turn hampers osteogenesis. Moreover, they found interactions between Msx2 and several PKC isoforms from different classes, which suggests a rather general inhibitory influence of PKC at least in these cells [66]. Admittedly, it is questionable whether results from trans-differentiating myoblasts can sufficiently depict the molecular mechanisms that occur during the osteogenesis of stem cells that naturally give rise to osteoblasts, but it still shows that PKC does not possess a ubiquitously definite role in this process.

These controversial results presume that the role of PKC in bone formation depends on the prevalence of other biological pathways in the cell, which vary between different cell types and might affect intracellular downstream targets of the kinase. The ambiguous role of PKC in osteogenesis is even better displayed in a single study with human BMMSCs that found an increased expression of the osteoblast marker osteocalcin, but a reduced expression of BMP2, after PKC inhibition [88].

### 4.2. Impact of Particular PKC Isoforms on Osteogenesis

However, the ambiguous involvement of PKC during osteogenesis becomes more enlightening when distinct isoforms or classes of the enzyme are investigated separately. In particular, in vitro experiments have mostly reported that specific inhibition of classical PKCs stimulates osteogenic differentiation [66,89,90,91], even more consistently when only regarding the isoforms PKCβ1/2 [91,92]. Nevertheless, PKCα has been connected with the osteogenesis of cultured cells partially positively [91,93,94] and partially negatively [36,90]. In addition, female mice with a homozygous deletion of the gene for PKCα displayed excessive bone formation [90].

Interestingly, the new isoform PKCδ has been concordantly shown to support osteogenic differentiation of stem/progenitor cells [89,91,95,96,97,98,99]. Additionally, mice with a homozygous PKCδ knockout exhibited deficiencies in embryonic osteogenesis [99]. In addition, the atypical isoform PKCζ has a positive role during the osteogenic differentiation of murine preosteoblasts [100]. It is remarkable that PKCβ and PKCδ have opposing roles for osteogenic differentiation, but both stimulate cell proliferation. This leads to the assumption that highly specific and time-resolved regulation of the different PKC isoforms is of great importance for proper bone formation. An overview of published studies that investigated the role of PKC or distinct isoforms during the osteogenic differentiation of stem/precursor cells with the principal outcomes can be found in Table 1.

### 4.3. Downstream Targets Mediating the Influence of PKC on Osteogenesis

While many studies provide evidence that PKC influences the expression of established osteogenic markers and the mineralization capability of differentiated cells, less is known about the direct molecular targets that build the molecular bridge between the kinase and successful osteogenesis. In addition to the already mentioned interactions with the transcription factor Msx2 [66], experiments by Kim et al. suggested that PKCδ phosphorylates Runx2, an important transcription factor for osteogenic differentiation, which might be a central reason why this isoform supports bone formation [104]. Beyond this, cell culture experiments have provided evidence that PKC interacts with the canonical Wnt pathway and that it affects the stability of the key transcription factor of this pathway, namely β-Catenin [105,106,107]. Furthermore, it has been shown that the kinase, especially PKCα, is able to phosphorylate β-Catenin at N-terminal serine residues, which leads to an enhanced depletion of the transcription factor [108], and that PKCε phosphorylates central amino acids of β-Catenin, which, in contrast, stabilizes the protein [106]. In addition to providing more striking examples that different isoforms partially have completely different functions, these studies provide distinct molecular mechanisms of how PKC impacts osteogenesis. However, there are still open questions regarding how exactly the canonical Wnt pathway affects bone formation afterwards and it must be kept in mind that this pathway is also controlled by many other factors that need to be considered and might themselves interact with PKC.

A third example of PKC-affected downstream targets, which in turn regulate osteogenesis, is the nuclear factor “kappa-light-chain-enhancer” of activated B cells (NF-κB) pathway. For example, in vitro experiments have shown that the two kinases protein kinase A (PKA) and PKC support the translocation of the transcription factor NF-κB from the cytosol into the nucleus and its binding to DNA [109]. Moreover, PKC isoforms from different classes were shown to regulate the activity of IκB kinase (IKK), which is an important upstream modulator of the NF-κB pathway [110,111]. The activated transcription factor NF-κB is primarily reported to inhibit the osteogenic differentiation of several stem/precursor cells of different origins [112,113,114,115].

These distinct molecular mechanisms between PKC and the control of osteogenesis have also been investigated in dental follicle cells (DFCs), which are ectomesenchymal precursor/stem cells with a broad differentiation potential [116,117]. Experiments have shown that an inhibition of classical PKC isoforms strongly supports the osteogenic differentiation of these cells by regulating the activity of the kinase Akt and subsequently affecting—at least partly via Akt—both canonical Wnt signaling and the NF-κB pathway. To be more concise, classical PKC inhibition enhanced Akt activity as well as nuclear expression of β-Catenin, while downregulating the expression of NF-κB and related proteins in osteogenically differentiating DFCs [101]. Beyond this, both nuclear β-Catenin and diminished NF-κB activity have been shown to support the osteogenic differentiation of DFCs [101,118,119], which presumes that those pathways contribute to the effect that classical PKCs (and Akt) exert on bone formation. Interestingly, the positive effect of PKCζ on osteogenic differentiation presumably also involves the activation of Akt [100]. In addition, experiments with DFCs have shown that the inhibition of classical PKCs downregulated the expression of sclerostin, which is predominantly known to impair osteogenic differentiation [102].

### 4.4. Endogenous Regulation of PKC During Osteogenesis

**Figure 3:**
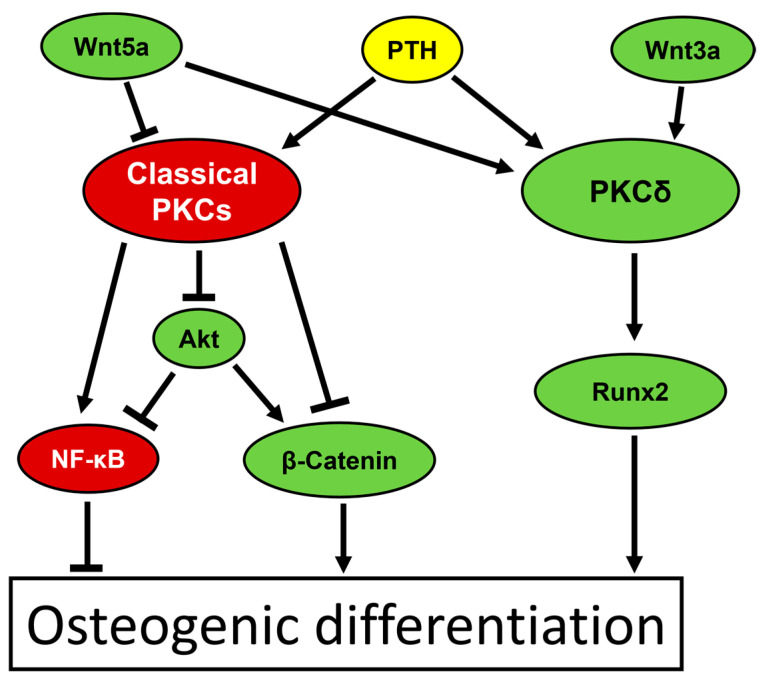
Schematic illustration of the molecular pathways that inhibit osteogenic differentiation of stem/precursor cells via classical PKCs or support it via PKCδ. Components which principally support osteogenesis are illustrated in light green, whereas components that inhibit it are shown in dark red. Because of its ambiguous role in osteogenic differentiation, PTH is depicted in yellow. For further explanations see text.

These observations lead to the assumption that classical PKC isoforms, on the one hand, and PKCδ, on the other hand, might serve as master regulators for osteogenesis with the former having a negative and the latter possessing a positive impact. In this context, it is further important how those isoforms themselves are endogenously regulated. Numerous pathways have been found to influence their activity. For instance, the osteo-promoting PKCδ can be activated by Wnt3a, which is known as an inducer of the canonical Wnt pathway [99,120], or by the parathyroid hormone (PTH) [95,121,122]. Interestingly, while the Wnt3a-induced mechanism depends on PLC activation, the pathway via PTH can either involve PLC or occur independently of it, although the latter is less understood to date [99,122]. However, PTH is also capable of activating classical PKC isoforms [123,124], which gives PTH an ambiguous role in osteogenic differentiation. Experiments with single cells have provided evidence that PTH administration can alter cellular calcium levels in different manners depending on the activity of other signaling proteins, which ultimately lead to completely different effects on osteogenic differentiation [125]. It is certainly conceivable that these different calcium responses might exert their contrary impacts on osteogenesis via the activation of different PKC isoforms. Another protein with an ambiguous role in PKC activation is Wnt5a, a mediator of non-canonical Wnt signaling, which is mostly reported to stimulate the enzyme in general but has been shown to inhibit classical PKC isoforms in experiments with DFCs. However, different in vitro studies agree that Wnt5a has a positive impact on osteogenic differentiation [78,101,126,127]. Moreover, experiments with endothelial progenitor cells have provided evidence that Wnt5a activates the isoform PKCδ [128], assuming that such an activation mechanism might also play a role during the differentiation of stem cells. Thus, Wnt5a probably supports osteogenesis by modulating the two antagonizing PKC axes contrarily. Figure 3 schematically illustrates those two axes, with either the osteo-inhibiting classical PKCs or the osteo-promoting PKCδ as key players, including the most relevant signaling pathways involved.

### 4.5. Impact of PKC on Bone Resorption

Notably, PKC not only affects osteogenesis, but also the contrary process bone resorption. General inhibition of PKC has been reported to disturb the receptor activator of nuclear factor-kappa B ligand (RANKL)-induced activity of osteoclasts, which suggests that PKC is necessary for the function of these cells [129,130]. In particular, the isoforms PKCβ2 and PKCδ as well as atypical isoforms were shown to be important for the differentiation and function of osteoclasts [131,132,133]. Since induction of osteolysis by RANKL includes activation of NF-κB [134], which hampers osteogenic differentiation (see above), the role of classical PKCs in bone metabolism appears to be predominantly catabolic.

## 5. Role of PKC During Adipogenic Differentiation

### 5.1. General Influence of PKC or Certain Isoforms During Adipogenesis

The impact of PKC on adipogenic differentiation is similarly complex, like its influence on osteogenesis. Interestingly, while these two processes are often regarded to be regulated contrarily [83,91,97], they can be induced in vitro—at least partly—by the same substances like, for example, insulin, insulin-like growth factor 1 (IGF-1) or insulin-like growth factor 2 (IGF-2), although higher concentrations of these substances are usually applied for adipogenic induction [135,136,137,138,139].

Studies that evaluated the general influence of PKC on adipogenesis have found inconsistent results [83,105,140,141,142,143,144], which assumes that different isoforms again have different impacts. Notably, investigation of classical isoforms also led to contradictory results [141,145]. In particular, PKCβ and PKCγ have been reported to promote adipogenesis [91,146], while PKCα rather hampers it [91,147,148]. Moreover, PKCδ—and especially particular splicing variants—were shown to be a negative regulator of adipogenesis in a number of studies [91,97,141,147,149], whereas another novel isoform, PKCε, supports it [150,151]. A very interesting observation is the overwhelming consensus about the role of PKCβ as an osteo-inhibiting and adipo-supporting enzyme, and that of PKCδ as osteo-supporting and adipo-inhibiting. Thus, balance between these two isoforms might substantially decide the fate of differentiating stem cells towards either adipogenesis or osteogenesis. At least in the case of PKCβ, this significance is likely limited to the earlier stages of differentiation, as McGowan et al. reported that expression of this isoform was no longer detectable in differentiated adipocytes in vitro. The same study found that, in contrast, the novel isoform PKCθ might play a pivotal role during late adipogenesis as its expression was highly induced in this period [148]. Ultimately, Table 2 provides an overview of the published studies that investigated the role of PKC or distinct isoforms during the adipogenic differentiation of different stem and precursor cells.

### 5.2. Endogenous Regulation of PKC During Adipogenesis

It is further interesting how PKC is endogenously regulated in cells during adipogenesis. For example, the canonical Wnt pathway and its effector protein β-catenin, which are regulated by PKC, are negatively associated with adipogenic differentiation of cultured preadipocytes [105]. In contrast, Keats et al. described that non-canonical Wnt signaling, induced by high glucose levels in this study, stimulates PKC and subsequently the adipogenic differentiation of BMMSCs [140]. This is surprising since the non-canonical Wnt pathway has also been reported to support bone formation (see above). However, the involved ligands differ in these two cases as osteogenesis was supported by Wnt5a, whereas adipogenesis was stimulated by Wnt11 [78,140]. Hence, although not conspicuous at first glance, the non-canonical Wnt pathway might have completely diverse effects on stem cell differentiation depending on its ligands—a pattern like the isoform-dependence of PKC.

### 5.3. Downstream Targets Mediating the Influence of PKC on Adipogenesis

A small number of studies further investigated the downstream mechanisms of PKC during adipogenic differentiation. For example, the pro-adipogenic effect of PKCε might arise from direct interactions with the transcription factor CCAAT/enhancer-binding protein β (C/EBPβ), which plays a crucial role during early adipogenesis, as these two proteins were found to be colocalized after adipogenic induction of mouse embryonic fibroblasts [150,153]. Moreover, experiments with preadipocytes by Gaillard et al. assumed that PKC might synergize together with PKA to enhance adipogenic differentiation [142], which shows that the effect of PKC activation on adipogenesis also depends on the activity of other cellular pathways.

## 6. Role of PKC During Chondrogenic Differentiation

### 6.1. General Influence of PKC or Certain Isoforms During Chondrogenesis

It is not surprising that studies on the general influence of PKC are also contradictory in terms of chondrogenic differentiation. For example, inhibition of the enzyme supported chondrogenesis in a study by Kulyk and Reichert, while hampering this process in experiments by Choi et al. Notably, both studies were carried out with cells from mesenchymal-derived tissues of chicken embryos [154,155]. A possible explanation for the controversial results was provided by experiments with human BMMSCs, in which cells were either cultured under high-glucose or low-glucose conditions. While the cells in high-glucose medium possessed a lower differentiation potential and a higher amount of phosphorylated PKC, it was the other way around under low-glucose conditions. Interestingly, chondrogenic potential could be increased by either inhibiting PKC in high-glucose BMMSCs or by stimulating the enzyme in low-glucose cells [156]. Thus, chondrogenic differentiation might proceed optimally with a moderate level of PKC activation where activity that is either too high or too low is counterproductive. It was further shown that general PKC inhibition blocked the proliferation of chondrogenic precursor cells [155]. However, proliferation plays a crucial role during cartilage formation and even in the development of many bones that arise from cartilage as a precursor in the process of endochondral ossification [157]. Hence, this strengthens the hypothesis that a certain minimum activity of PKC is certainly required for chondrogenesis.

Isoform-specific evaluation showed that PKCα, PKCγ and PKCε were upregulated during chondrogenic differentiation of mesenchymal chicken cells and that PKCα in particular actively supports it [155,158,159,160]. Moreover, atypical PKCs have been shown to be constitutively expressed during chondrogenic differentiation [155], but it remains unclear if they essentially participate in this process. The regulation pattern of the isoforms does not stand in contrast to the pattern during osteogenic differentiation, although the latter might be especially dependent on the additional activation of PKCδ (see above). Therefore, during endochondral ossification, the activation of PKCδ might serve as a switch after chondrogenesis to initiate subsequent bone formation. However, basal PKCδ activity is possibly also a prerequisite for chondrogenesis as downregulation of this enzyme reduced cartilage formation in a study with mesenchymal cells from chicken [161].

### 6.2. Endogenous Regulation of PKC During Chondrogenesis

Upstream, several extracellular proteins have been described that regulate the chondrogenic differentiation of cells via PKC-involving pathways. Only some of them will be mentioned here, with a focus on those that were already discussed for their role during osteogenic and/or adipogenic differentiation. Interestingly, while IGF-1 and Wnt5a both stimulate the chondrogenic differentiation of mesenchymal cells from chicken embryos via PKCα activation [162,163], PTH was found to inhibit the terminal differentiation of adult human and embryonic chicken chondrocytes [164,165]. Reverting to the example of endochondral bone formation, it thus might be pivotal to suppress PTH signaling at least in the earlier phases, while continuous activity of both IGF-1 and Wnt5a keeps the process running. Moreover, intracellular signals might also modulate chondrogenic differentiation. For example, Lim et al. showed that the pharmacological disruption of the actin cytoskeleton supports the chondrogenic differentiation of mesenchymal cells from chicken embryos via PKCα activation [160].

### 6.3. Downstream Targets Mediating the Influence of PKC on Chondrogenesis

Another interesting aspect is how PKC ultimately controls chondrogenic differentiation in the cell. Previous research was especially focused on the mitogen-activated protein kinase (MAPK) family, which plays a central role in cartilage formation [166]. More precisely, the supporting effects of PKC on chondrogenesis are considered to be exerted—at least partly—via the regulation of Erk1, a member of the MAPK family, which disturbs the differentiation process of cultured mesenchymal cells from chicken embryos [158,167]. Moreover, a study by Yoon et al. with rabbit chondrocytes even suggests that Erk inhibition is pivotal to obtain and maintain a chondrogenic phenotype including typical characteristics like enhanced collagen II expression [168]. By contrast, another MAPK protein, namely the kinase p38, supports chondrogenic differentiation of embryonic chicken cells and its activity is probably also affected by PKC. However, while the published studies generally agree on the chondro-supportive effect of p38, it is controversial regarding the possible involvement of PKC, which has been described in different studies to either stimulate p38, have no effect on its activation or even inhibit it under certain circumstances [158,165,169,170].

## 7. Role of PKC During Differentiation into Other Tissue Cells

### 7.1. Role of PKC During Neuronal Differentiation

Interestingly, various stem cells derived from non-neural origins like MSCs have been demonstrated to be able to trans-differentiate into neuron-like cells in vitro under certain culture conditions and it appears very likely that PKC might be of great significance in these processes too. For example, a study with human dental pulp stem cells provided evidence that stimulation by 12-O-tetradecanoylphorbol 13-acetate, which mimics DAG and thus activates classical and novel PKCs [171], together with the simultaneous stimulation of cellular cAMP levels, constitute pivotal factors that induce the cells to differentiate towards neurons—although admittedly, additional (pre-)treatment with growth factors is further required [172]. In contrast, classical and novel PKCs have been negatively associated with the neuronal trans-differentiation of MSCs from human umbilical cords [173], which hints again at the relevance of other cellular pathways that might interact with PKC and modulate its impact on differentiation. Isotype-specific investigations have predominantly revealed a positive impact of novel isoforms. In particular, the expression of PKCδ has been shown to rise significantly during rat brain development [174] and the expression of PKCε increases after neuronal induction of human BMMSCs [175]. Moreover, the expression of PKCη was induced during neuronal trans-differentiation of ADSCs [176]. In addition, experiments with other progenitor cells have shown that the classical isoforms PKCα and PKCγ, as well as the two atypical isoforms, are positively associated with neuronal differentiation [177,178,179]. Thus, stimulating these isoforms might be beneficial in attempts to trans-differentiate non-neural stem cells into neuron-like cells. However, it remains elusive how PKC or certain isoforms ultimately control neurogenesis and whether the importance of the different isoforms depends on the origin of the stem cell.

### 7.2. Role of PKC During Differentiation into Keratinocytes and Cardiomyocytes

In addition, the role of PKC has already been investigated during the differentiation of several more tissue cells, although usually restricted to only a few distinct isoforms. For example, PKCα und PKCη were shown to support the terminal differentiation of isolated keratinocytes, which is accompanied by a proliferation stop [180,181,182]. Notably, these studies presume that PKCα exerts its influence on keratinogenesis primarily via the regulation of cell cycle-associated proteins like p21 and p53, resulting in cell cycle arrest, which might trigger the terminal differentiation of keratinocytes [180,181].

As another example, the novel isoforms PKCδ and PKCε have been implicated in improving the cardiomyogenic differentiation of MSCs [183,184]. Notably, pharmacological PKC stimulation with phorbol myristate acetate, which is another substance that mimics DAG and thus activates classical and novel PKCs [185], has already successfully been used to differentiate MSCs into functional cardiomyocytes and, moreover, such cells have exhibited promising therapeutic potential after transplantation into an infarcted heart in a rat model [186,187].

## 8. Conclusions and Outlook

The numerous examples have shown that PKC possesses a central role during the differentiation of stem and precursor cells into various specialized tissue cells, while its influence highly depends on the observed isoform. Table 3 provides a summarizing overview of the principal effect of each isoform on the differentiation into osteoblasts, adipocytes, chondrocytes or neurons.

The central role of PKC makes the enzyme a promising target to improve regenerative therapies, where stem cells are used to restore damaged tissue. However, the impact of PKC on the differentiation of stem cells into specialized tissue cells is—at least partially—inconsistent. In some cases, even the same isoform might impact a certain process differently depending on several conditions like the activity of other cellular pathways, which is best exemplified by the role of PKCα during osteogenesis. This would presumably also apply when attempting to treat patients in regenerative therapies by targeting PKC. Given the assumption that the cellular activity of many biological pathways varies between different patients, this would highly affect the therapy outcome. However, there is also a certain consensus in published studies regarding the role of at least some isoforms in a certain process like the overall positive impact of PKCδ on osteogenic differentiation or that of PKCβ on adipogenesis. Because of their decisive role in the corresponding differentiation pathways, it is probably best to focus on these isoforms for therapeutic approaches.

Another factor that must be kept in mind when manipulating PKC for regenerative purposes in humans is that the enzyme not only affects differentiation but also other important biological processes like proliferation and apoptosis. Consequently, possible side effects need to be strictly considered to prevent undesired events like uncontrollable cell division, which might ultimately lead to cancer. Thus, a focus on only one isoform would certainly help not only to achieve consistent desired results but would also reduce possible side effects. However, pharmaceutical agents targeting PKC like phorbol esters bear the restriction that they usually affect more than only one isoform. Instead, the rapidly emerging gene editing techniques might be a promising tool for regenerative therapies in the future with the ability to accurately up- or downregulate expression of a certain isoform.

Moreover, endogenous control of PKC activity, especially under varying conditions, as well as downstream targets mediating its influence on the differentiation of stem/precursor cells into functional tissue cells are still poorly evaluated. Understanding how, for example, PKCδ supports osteogenesis will not only provide further insights into the regulatory mechanisms but also reveal new targets, whose manipulation would probably enable even more precise control of the differentiation process.

## Figures and Tables

**Table 1:** Regulation of the osteogenic differentiation of stem/precursor cells by PKC.

Cells	Isoforms	Impact on Osteogenic Differentiation		Ref.
ADSCs (human)	General	+	WNT5A induced PKC activity as well as osteogenic markers and mineralization	[78]
ADSCs (human)	General	+	PKC overactivation supported differentiation unless cells were pretreated with a PKC inhibitor	[83]
BMMSCs (human)	General	+	N-terminal amelogenin peptide induced both PKC activity and osteogenic differentiation	[79]
ADSCs (mouse)	General	+	Overexpression of miR-26a-5p inhibited both osteogenic differentiation and phosphorylation of PKC	[80]
BMMSCs (mouse)	General	+	Inhibition of cystathionine-β-synthase inhibited both the osteogenic differentiation and expression of phosphorylated PKC	[81]
Bone marrow stroma cells M2-10B4 (mouse)	General	+	Osteogenic markers were inhibited by PKC inhibitors	[82]
Myoblasts C2C12 (mouse)	General,	−	Inhibition of classical PKCs and general PKC inhibition induced osteogenic markers, while PKC overactivation inhibited osteogenesis	[66]
	classical PKCs	−		
BMMSCs (human)	General	+/−	Inhibition of PKC inhibited osteocalcin expression but increased BMP2 expression	[88]
DFCs (human)	Classical PKCs	−	Classical PKCs were downregulated during osteogenic differentiation; inhibition of classical PKCs stimulated mineralization	[101]
DFCs (human)	Classical PKCs	−	Inhibition of classical PKCs stimulated mineralization, which was impaired by treating cells with the protein sclerostin; expression of sclerostin was downregulated after inhibition of classical PKCs	[102]
BMMSCs (human)	Classical PKCs,	−	Inhibition of classical PKCs stimulated osteogenic markers and mineralization; inhibition of PKCδ hampered activity of alkaline phosphatase	[89]
	PKCδ	+		
BMMSCs (human and mouse)	PKCα	+	Overexpression of PKCα induced osteogenic markers (human and murine BMMSCs) and mineralization (murine BMMSCs)	[93]
Embryonic fibroblasts C3H10T1/2 (mouse)	PKCα	+	Inhibition of PKCα hampered osteogenic markers and mineralization	[94]
Osteogenic precursor cells MC3T3-E1 (mouse)	PKCα	−	Downregulation of PKCα supported osteogenic differentiation	[90]
BMMSCs (human)	PKCα,	+/−	Co-cultivation of BMMSCs and myeloma cells inhibited both mineralization and expression of phosphorylated PKCα und PKCδ, but increased expression of phosphorylated PKCβ1; inhibition of classical PKCssupported mineralization	[91]
	PKCβ1,	−		
	PKCδ	+		
Periodontal ligament stem cells (human)	PKCβ2	−	Decreased expression of phosphorylated PKCβ2 was associated with increased expression of osteogenic markers	[92]
ADSCs (human)	PKCδ	+	Inhibition of PKCδ hampered osteogenic markers	[95]
BMMSCs (human)	PKCδ	+	Inhibition of PKCδ hampered Jagged-1-induced osteogenic differentiation	[96]
BMMSCs (human)	PKCδ	+	PKCδ was induced during osteogenic differentiation; inhibition of PKCδ hampered osteogenesis	[97]
ADSCs (mouse)	PKCδ	+	Oncostatin M stimulated both osteogenic differentiation and activity of PKCδ; downregulation of PKCδ inhibited osteogenesis	[98]
Bone marrow stroma cells ST2 (mouse)	PKCδ	+	Downregulation of PKCδ inhibited WNT3A-induced osteogenic differentiation	[99]
Osteogenic precursor cells MC3T3-E1 (mouse)	PKCη,	+	Expression of PKCη was associated with expression of osteogenic markers; expression of PKCθ was downregulated following osteogenic induction	[103]
	PKCθ	−		
Osteogenic precursor cells MC3T3-E1 (mouse)	PKCζ	+	PKCζ was activated after osteogenic induction and supported osteogenesis by phosphorylating vimentin	[100]

Note: ‘+’ indicates a supportive role, while ‘−’ indicates an inhibiting role of the corresponding PKC isoform(s) for osteogenic differentiation.

**Table 2:** Regulation of the adipogenic differentiation of stem/precursor cells by PKC.

Cells	Isoforms	Impact on Adipogenic Differentiation		Ref.
BMMSCs (human)	General,	+	PKC activity was induced during adipogenic differentiation; general PKC inhibition and specific inhibition of PKCε hampered adipogenic markers	[140]
	PKCε	+		
Embryonic fibroblasts 3T3-L1 (mouse)	General,	+	General PKC inhibition and specific inhibition of classical PKCs hampered adipogenic differentiation; inhibition of PKCδ supported adipogenesis	[141]
	classical PKCs,	+		
	PKCδ	−		
Embryonic fibroblasts 3T3-L1 (mouse)	General	+	PKC inhibition hampered adipogenic differentiation	[105]
Adipogenic precursor cells Ob1771 (mouse)	General	+	PKC overactivation supported adipogenesis when cells were simultaneously treated with substances that enhance cAMP concentration	[142]
ADSCs (human)	General	−	PKC overactivation inhibited adipogenic differentiation unless cells were pretreated with a PKC inhibitor	[83]
Embryonic fibroblasts 3T3-L1 (mouse)	General	−	PKC inhibition supported adipogenic differentiation	[143]
Adipogenic precursor cells (rat)	General	−	PKC inhibition supported adipogenic differentiation	[144]
Embryonic fibroblasts 3T3-F442A (mouse)	PKCα,	−	Expression of PKCα und PKCδ were reduced during adipogenic differentiation; downregulation of PKCγ and PKCε inhibited adipogenesis	[147]
	PKCδ,	−		
	PKCγ,	+		
	PKCε	+		
Embryonic fibroblasts 3T3-L1 (mouse)	PKCα	−	Expression of PKCα was downregulated during adipogenesis; expression of PKCβ was temporarily induced during differentiation, but declined at later periods; PKCθ was detected only in differentiated adipocytes	[148]
	PKCβ	+/−		
	PKCθ	+		
Embryonic fibroblasts 3T3-L1 (mouse)	PKCα	−	Phosphorylation of PKCα was associated with inhibition of adipogenesis after treatment with evodiamine	[145]
BMMSCs (human)	PKCα,	+/−	Co-cultivation of BMMSCs and myeloma cells supported adipogenesis and enhanced the expression of phosphorylated PKCβ1 while inhibiting the expression of phosphorylated PKCα und PKCδ; the inhibition of classical PKCs hampered adipogenic differentiation	[91]
	PKCβ1,	+		
	PKCδ	−		
ADSCs (human)	PKCβ	+	Activation of PKCβ was associated with induction of adipogenic differentiation by atypical antipsychotics; inhibition of PKCβ hampered adipogenesis	[146]
BMMSCs (human)	PKCδ	−	Inhibition of PKCδ induced adipogenic differentiation	[97]
Embryonic fibroblasts 3T3-F442A (mouse)	PKCε	+	Adipogenic differentiation stimulated expression of PKCε; overexpression of PKCε supported adipogenesis	[150]
Embryonic fibroblasts 3T3-L1 (mouse)	PKCε	+	Adipogenic differentiation was supported by PKCε stimulation and inhibited by PKCε downregulation	[151]
Adipogenic precursor cells (rat)	PKCζ	+	Expression of PKCζ in the cytoplasm was enhanced during adipogenic differentiation; insulin treatment increased expression of PKCζ in the cytoplasm, plasma membrane and nucleus	[152]
Fetal brown adipocytes (rat)	PKCζ	+	Activation of PKCζ was associated with IGF-1-induced adipogenic differentiation	[135]

Note: ‘+’ indicates a supportive role, while ‘−‘ indicates an inhibiting role of the corresponding PKC isoform(s) for adipogenic differentiation.

**Table 3:** Summary of the role of distinct PKC isoforms during multilineage differentiation of stem/precursor cells.

Group	Isoform	Impact on…			
		Osteogenic Differentiation	Adipogenic Differentiation	Chondrogenic Differentiation	Neuronal Differentiation
Classical PKCs	α	+/−	−	+ +	+
	β	−	+ +	?	?
	γ	?	+	+	+
Novel PKCs	δ	+ +	− −	+	+
	ε	+	+	+	?
	η	+	?	?	+
	θ	−	+	?	+
Atypical PKCs	ζ	+	+	?	+
	ι	?	?	?	+

Note: ‘+’ indicates a (highly for double +) supportive role, ‘−’ indicates a (highly for double −) inhibiting role, ‘?’ indicates that no distinctive role could be allocated to date.

## Abbreviations

ADSCs	adipose-derived stem cells
AMPK	AMP-activated protein kinase
BMMSCs	bone marrow-derived mesenchymal stem cells
BMP2	bone morphogenetic protein 2
C/EBPβ	CCAAT/enhancer-binding protein β
DAG	diacylglycerol
DFCs	dental follicle cells
GPCRs	G protein-coupled receptors
IGF-1	insulin-like growth factor 1
IGF-2	insulin-like growth factor 2
IKK	IκB kinase
IP_3_	inositol 1,4,5-trisphosphate
MAPK	mitogen-activated protein kinase
MSCs	mesenchymal stem cells
Msx2	Msh homeobox 2
NF-κB	nuclear factor “kappa-light-chain-enhancer” of activated B cells
PB1	Phox and Bem1
PDK1	3-phosphoinositide-dependent protein kinase-1
PKA	protein kinase A
PKC	protein kinase C
PLC	phospholipase C
PTH	parathyroid hormone
RANKL	receptor activator of nuclear factor kappa B ligand
RTKs	receptor tyrosine kinases
